# Reprogramming of fibroblast cells to totipotent state by DNA demethylation

**DOI:** 10.1038/s41598-023-28457-8

**Published:** 2023-01-20

**Authors:** Mohammad H. Ghazimoradi, Kouichi Hasegawa, Ehsan Zolghadr, Samaneh Montazeri, Shirin Farivar

**Affiliations:** 1 Faculty of Life Sciences and Biotechnology, Shahid Beheshti University, Tehran, Iran; 2 Institute for Integrated Cell-Materials Science, Kyoto University, Kyoto, Japan; 3 Institute for Stem Cell Biology and Regenerative Medicine, Bangalore, India; 4 Department of Physics and Astronomy, University of Alabama, Tuscaloosa, AL 35487 USA; 5 Nanobiotechnology Research Center, Avicenna Research Institute, ACECR, Tehran, Iran

**Keywords:** Epigenetic memory, Pluripotency, Reprogramming, Stem cells

## Abstract

Many attempts have been made to induce high-quality embryonic stem cells such as pluripotent stem cells and totipotent stem cells, but challenges remain to be overcome such as appropriate methods and sources. Demethylation of the genome after fertilization is an important step to initiate zygote gene activation, which can lead to the development of new embryos. Here, we tried to induce totipotent stem cells by mimicking DNA demethylation patterns of the embryo. Our data showed, after induction of DNA demethylation via chemicals or knockdown of Dnmts, cells positive for Nanog, and Cdx2 emerged. These cells could differentiate into the pluripotent and trophoblast lineage cells in-vitro. After transferring these cells to the uterus, they can implant and form embryo-like structures. Our study showed the importance of DNA demethylation roles in totipotent stem cell induction and a new and easy way to induce this cell type.

## Introduction

Totipotent stem cells have the best potential for clinical and research usage among other stem cells since they can differentiate into all cell types and provide an outstanding model for studying the development of preimplantation and postimplantation stages. However, their usage is hindered due to a lack of an appropriate source^1^. Many researchers devote their time to dedifferentiating somatic cells into stem cells with high potency, including cells with toti or pluri potency^2,3^. Approaches for this reprogramming are nucleus transfer, genetic manipulation, and cell fusion to induce early embryonic phenotypes. Unfortunately, these methods are invasive and have low efficiency^2–4^. It is shown that the isolation and maintenance of these cells are not promising for ethical problems and a low number of these cells. In a culture of embryonic stem cells (ES cells), 2C phenotypes have been observed, but their low-frequency patterns are not repeatable and cannot be used and maintained^5,6^. In the most recent research, the co-culture of trophoblast and ES cells could form blastocyst-like structures. Although this study has shown a strong potential for inducing artificial developmental stages, the induced structures could not develop live offspring and could not offer totipotent stem cells^7^. Despite all the limitations on the induction and isolation of totipotent stem cells, there is no consistent model for the mechanism of totipotent state initiation^8^. It has been shown that the knockdown of epigenetic and structural-related genes could efficiently increase the number of 2C cells in ESC cultures such as CTCF^9^, HDACs^10^, LSD1^11^, and many more which has been reviewed comprehensively^12^. However, this induction method could be an enhanced opportunity for increasing the number of 2C cells in ESC culture. Induction of 2C or 4C-like cells has also been done with chemical inhibitors, which could promote totipotent cells in ESC culture. This method could also be the promotion of 2C cells in ESC culture^13^.

Methylation of DNA, which occurs on cytosine, is one of the most important epigenetic modifications^14,15^. This modification directly regulates gene expression. Over half of the genes in the genome have CpG islands that show the crucial role of this modification in establishing transcription patterns^16,17^. After fertilization, DNA methyltransferases (Dnmt 1, 3A, and B) will not be effective in the nucleus^18,19^ and will relocate to the cytosol. This relocation causes demethylation of the zygote by destabilizing and diluting of DNA methylation, which is called passive demethylation. It has been shown that many embryonic and organogenesis genes, which have been hypermethylated in postimplantation stages, are controlled and upregulated by demethylation^20,21^. It is noteworthy that the induction of demethylation can artificially dedifferentiate many cells into a stem cell lineage^22–27^. In conclusion, global DNA demethylation of zygotes in the early stages of development^28,29^, overexpression of embryogenesis-related genes after DNA demethylation^30^, and many studies show the administration of demethylation agents (mostly 5-Azacitidine (Aza)) leads to induction of stem cells, especially ICM and trophoblast stem cells^30–35^ might be evidence to show the role of global DNA demethylation in the induction of totipotent stem cells. In addition, it has been shown that DNMTs could inhibit ZSCAN4^36,37^, a co-effector of DUX and totipotent stem cells^38,39^. DNMTs also regulate TERT and telomeres^40^ which show a significant role of Dnmts in the totipotent state. Based on these notions, we tried to induce totipotent stem cells from fibroblast cells. Our data showed that we reprogram Nanog and Cdx2 positive cells by genome demethylation. Transferring these induced totipotent (iTot) cells directly to the uterus of pseudopregnant mice. After investigation, the development of these cells into embryo-like structures was detected.

## Results

### Aza could induce Nanog and Cdx2 positive cells which expressed MERVL

**Figure 1:**
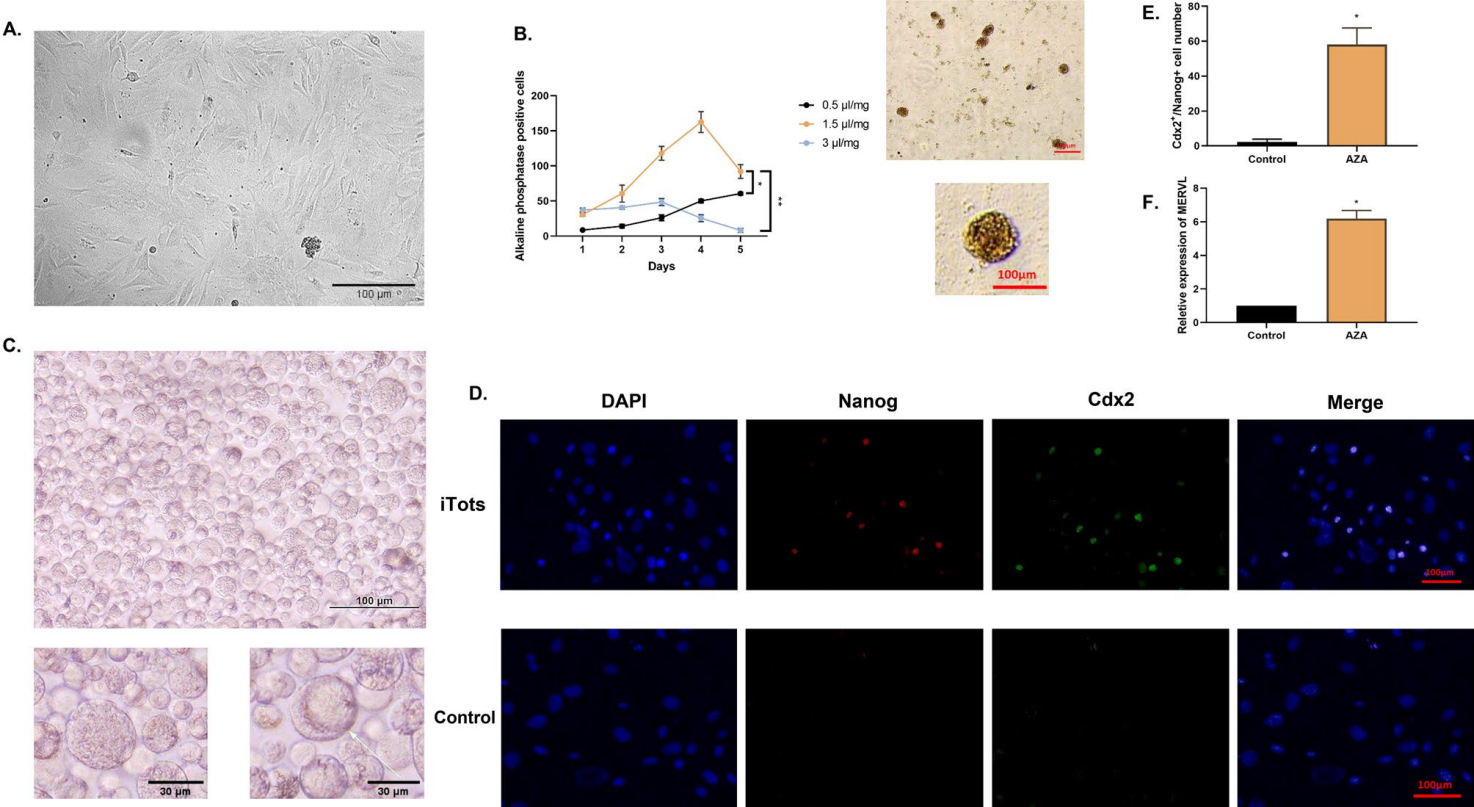
Induction of totipotent stem cells. (**A**) Fibroblast extraction is used as a source due to easy handling. (**B**) Aza could induce alkaline phosphatase positive cells (*P < 0.05, **P < 0.01). (**C**) Induced cells after sorting show significant morphologies. (**D**) Aza administration on fibroblast cells leads to Nanog and Cdx2-positive cells. (**E**) Number of Nanog and Cdx2 positive cells are significantly higher in Aza-treated cells in their absence in the control group (*P < 0.05). (**F**) MERVL induced in AZA-treated cells versus untreated cells (*P < 0.05).

**Figure 2:**
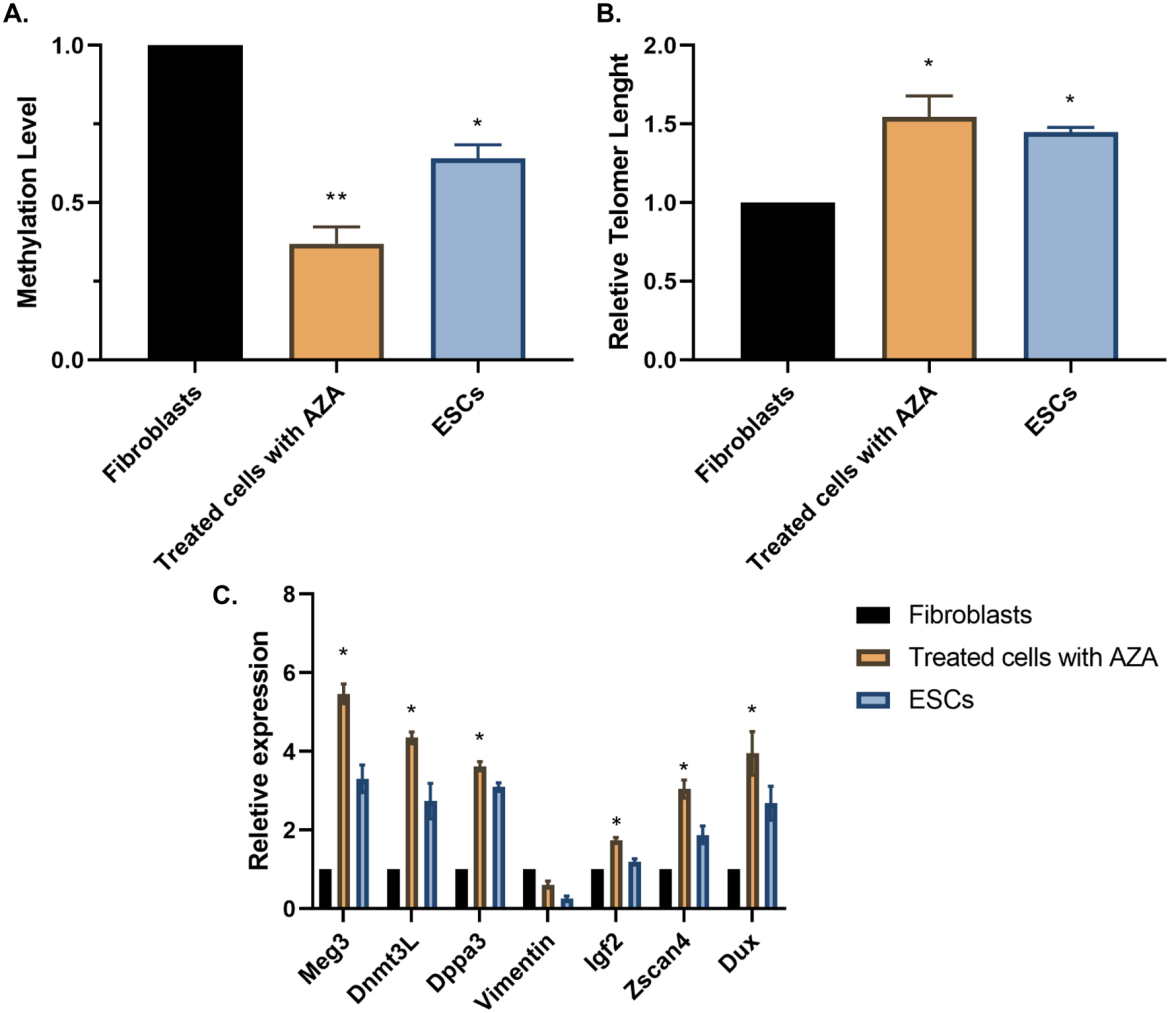
Molecular characterization of iTots shows overexpression of totipotent-related genes. (**A**) Aza-treated cells have lower DNA methylation levels in sorted cells versus untreated cells (*P < 0.05, **P < 0.01). (**B**) Telomere of Aza-treated cells increases in comparison with fibroblasts (*P < 0.05). (**C**) Gene expression analysis of iTots show their potential totipotent state comparing with fibroblasts and ESCs (*P < 0.05).

Fibroblast cells were chosen due to their transcription network and easy handling. Cells have been treated with different doses of Aza to identify the best dose (Fig. 1A,B). Administration of one dose of Aza in any concentration cannot lead to a high number of alkaline phosphatase-positive cells, which is a marker of embryonic stem cell proliferation. However, after treating cells with one and a half micrograms per milliliter of Aza for three days, results in a high number of alkaline-positive cells are identified (Fig. 1B). We use the differential affinity of induced cells versus fibroblast to sort the induced cells as this method is done in the isolation of embryonic stem cells from MEFs. Briefly, after trypsinization, the cells are transferred to new dishes. Fibroblast cells have a stronger affinity for culture dishes and attach sooner than induced cells. Therefore, transferring media containing iTot cells after the attachment of fibroblast can lead to cell sorting. All of the further experiments from here are carried out on sorted cells. After treating cells with Aza, some structures can be seen in isolated cells. There were small, large, and large cells with cavities that could be seen in isolated cells (Fig. 1C). Afterward, the presence of ICM marker Nanog and trophectoderm (TE) marker Cdx2 are examined by immunofluorescence. These markers have an important role in early embryonic development and embryogenesis and are considered 4C cell markers. We also checked the MERVL expression in cells. Isolated cells were positive for Cdx2 and Nanog, and expression of MERVL (as a marker of totipotent stem cells) has increased (Fig. 1D,F). The measurement of DNA methylation of these cells shows increased demethylation of the genome, which is the main alteration in this study for the induction of these structures (Fig. 2A). It also has been shown that administration of Aza could result in the down-regulation of trophoblast and pluripotency genes in treated cells which results in overexpression of these genes^33,34^. These data are consistent with the genome demethylation of totipotent stem cells and Aza-treated cells. Interestingly, after passaging cells, we do not recognize any aging-related signs, despite the differentiation of these cells in the cell culture dish. Due to this observation, the telomere length of treated cells on different days is measured and it keeps increasing on the sixth day (Fig. 2B). Demethylation can disrupt imprinting patterns. The upregulation of imprinting modulators or unchanged expression of imprinting genes can be a demonstration of methylation pattern status as well as a sign of totipotency which has been reported previously (12). Dnmt3l, Dppa3, Meg3, and Igf2 genes are selected for this reason. The results of Meg3, Dnmt3l, and Dppa3 genes which also have higher expression iTot cells versus other groups (Fig. 2C). Upregulation of these genes indicates a specific effect of genome demethylation. Indeed, expression of the Igf2 gene in imprinting loci is not significantly different between groups. We measured Vimentin expression in different groups to investigate the fibroblast-specific expression genes. In iTot cells, the expression of vimentin is decreased compared to the fibroblast cells. Zscan4 and Dux (totipotent stem cell marker and transcribtion factors) also have been investigated. Our data showed cells treated with Aza have higher expression of Zscan4 and Dux versus fibroblasts and ESCs (Fig. 2C).

### Dnmt1 and Dnmt3A could mimic the role of Aza in fibroblast cells

**Figure 3:**
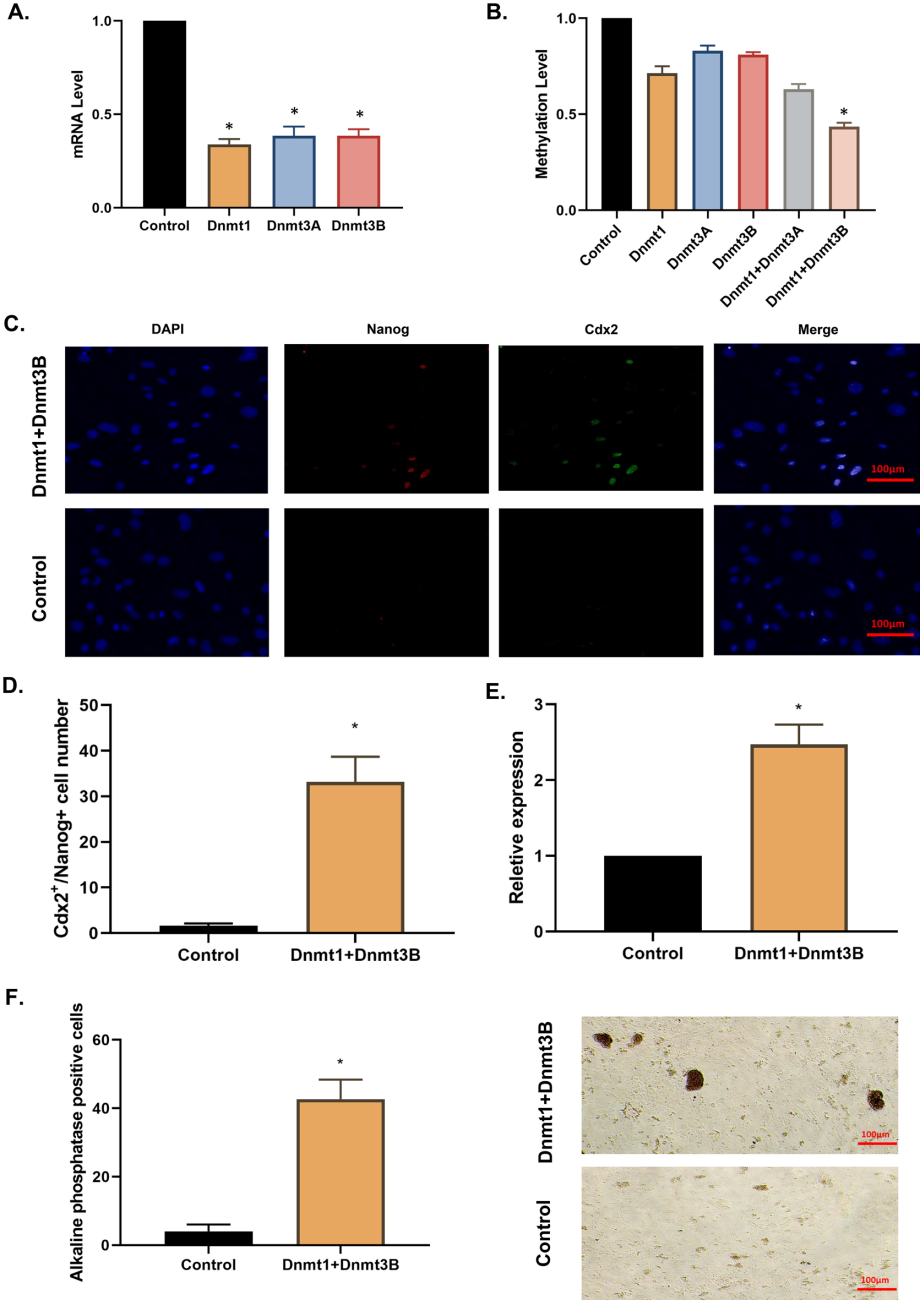
Dnmt1 and Dnmt3B could induce a totipotent state in fibroblast. (**A**) Dnmt1, Dnmt3A, and Dnmt3B result in efficient reduction of the mRNA level of these genes (*P < 0.05 for all groups versus treated cells with scramble control). (**B**) Dnmt1 and Dnmt3B knockdown results in significant DNA demethylation in comparison with control group (*P < 0.05). (**C**) Knockdown of Dnmt1 and Dnmt3B results in induction of Nanog and Cdx2 positive cells. (**D**) Number of Nanog and Cdx2 positive cells in the Dnmt1 and Dnmt3B Knockdown group is significantly higher in the control group which is almost complete absence of these cells (*P < 0.05). (**E**) Overexpression of MERVL detected in absence of Dnmt1 and Dnmt3B versus non treated fibroblast (*P < 0.05). (**F**) Cells treated with Dnmt1 and Dnmt3B show alkaline phosphatase activity (*P < 0.05).

Aza could induce DNA demethylation by targeting DNMTs. In this essence, we hypothesized that the knockdown of these genes could be responsible for induced totipotent stem cells. So, we knock down Dnmt1 and Dnmt3A, which are major Dnmt proteins with methylation activity, and analyze the methylation level of the genome (Fig. 3A,B). Dnmt1 and Dnmt3A co-inhibition results in genome-efficient hypermethylation, activation of MERVL, and the presence of Nanog and Cdx2 positive cells (Fig. 3C–E). These cells were also positive for alkaline phosphatase (Fig. 3F).

### Induced totipotent stem cells have developmental potential

**Figure 4:**
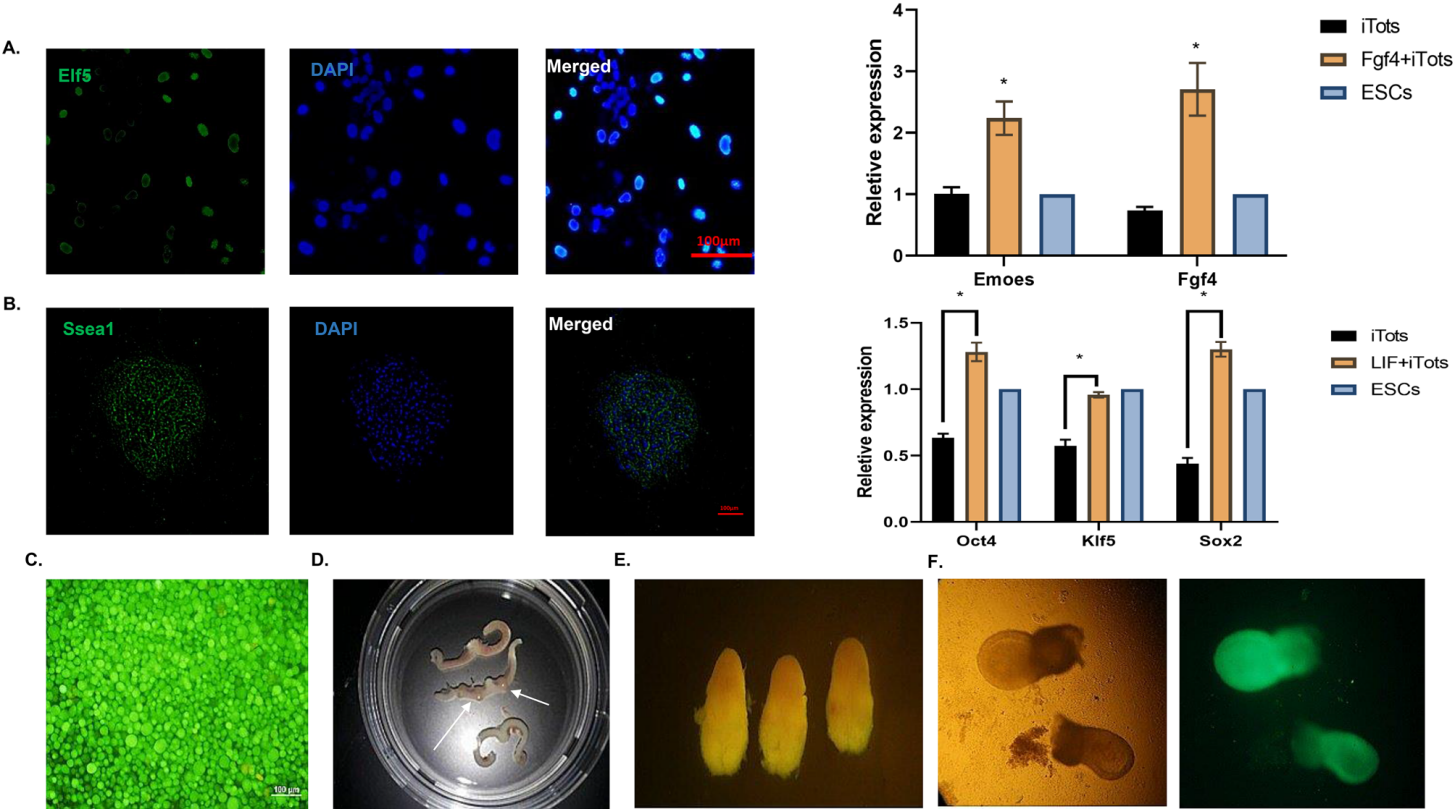
Induced totipotent stem cells show developmental potential. (**A**) ITot cells could be differentiated into trophoblast cells and have higher expression of trophoblast-related gene expression (*P < 0.05). (**B**) ITot cells could be differentiated into pluripotent stem cells and have higher expression of pluripotent-related gene expression (*P < 0.05). (**C**) Induced GFP-positive cells treated with Aza. (**D**) Transfer of Induced GFP-positive cells results in the implantation of cells. (**E**) Transferred cells could develop sacs in the uterus. (**F**) Transferred cells could develop embryo-like structures.

After obtaining these results from molecular tests, the functional test is crucial. In this essence, in-vitro differentiation of induced cells into ICM and Trophoblast lineage cells seems necessary. For these purposes, induced cells are cultured in a medium containing LIF and bFGF. The culture of induced cells with LIF results in pluripotent stem cell colonies that are positive for Ssea1 and overexpression of pluripotency-related genes as expected. On the other hand, bFGF-treated cells could lead to trophoblast morphologies that are positive for Elf5 and have upregulated trophoblast-related genes (Fig. 4A,B). In-vivo tests for the evaluation of the full potential of these cells were conducted. At first sight, often-used chimerism testing is supposed to be a good candidate, but due to the large diameter of induced structures, whole structures are injected into pseudopregnant mice. In this regard, we inject GFP-positive iTot cells (Fig. 4C). After injection, ten DPC mothers are dissected (Fig. 4D). The uterus of dissected mice carried sacs (Fig. 4E) containing eight DPC embryo-like structures that were positive for GFP (Fig. 4F).

## Discussion

In brief, we report the reprogramming of fibroblast cells to a totipotent state. These cells demonstrate morphology near the normal totipotent stem cells. Most features of these cells have the potential to implant, form, and grow embryo-like structures. However, its genome-scale investigation remains to be elucidated. This research shows the role of demethylation in the initiation of ZGA, induction of totipotency, and its possible role in the initiation of embryo development programming.

In this study, Cdx2 and Nanog are chosen as markers to determine embryonic fate. Cdx2 and Nanog are the markers of the trophoblast and epiblast cells and are the main difference between 2 and 4C cells. We determine the expression of related imprinting genes for investigating the reprogramming process. We chose Dppa3 and Dnmt3L, which have great roles in maintaining and establishing the right imprinting pattern, in agreement with a natural process; and we observe the same expression pattern. Apart from the expression of these genes, we investigate the expression of Meg3, which shows its relation to successful chimera engrafting. These data show that our phenotypes have the potential for chimera engraftment. Based on Meg3 results we performed an embryo transfer test which was successful. This data showed that DNA demethylation could induce a totipotent state and validate its role as a determining factor in the development of embryos and zygotes. This reprogramming is based on using one well-known chemical substrate that is labor free and could be done on finally differentiated cells such as fibroblast in comparison with the latest advances which use ESC as the source. Finally, iTot cells could form post-implantation embryos which is an advancement for prior researchers.

## Materials and methods

### Cell culture

For fibroblast isolation, the ear of the mouse (BALB/c) was minced. The pieces were washed with 75% methanol for two minutes. Next, ethanol was removed by washing with DMEM (Gibco). The next step was to chop the pieces with a surgical blade and trypsinize them at 37 C. The solution was centrifuged and re-suspended in DMEM containing 10% FBS (Invitrogen) and 2% pen-strep (Invitrogen). When the cells reach 70–80% percent confluency, they have been trypsinized and passaged. For treatment, the medium was changed when the cells reached 75–80% confluency, and after two hours, 1.5 µg per milliliter of 5-AzaC (sigma-A2385-100MG) was added. 5-AzaC was dissolved in the culture medium and freshly used every day up to the third day. For the extraction of ES cells, the mice were mated overnight. We let sufficient time pass and then dissected the mice, and 3 DPC embryos were transferred into a plate with a full medium consisting of high glucose DMEM, 103 units per milliliter of LIF (Invitrogen), and 10% of FCS (Invitrogen). Regarding the lineage tracing test, GFP-positive transgenic mice (BALB/c) were provided by the Royan Institute. 103 U/ml and 25 ng/ml of LIF and bFGF (Sigma) were added to iTot cells to differentiate them into pluripotent stem cells and trophoblast lineages.

### Alkaline phosphatase assay

To stain cells for alkaline phosphatase, the medium of cells has been aspirated and cells have been washed with PBS. Furthermore, cells were fixed with ice methanol and washed with PBS again. Afterward, cells have been stained with a Sigma Alkaline phosphatase kit. The A and B solutions have been mixed and added to cells. Cells have been washed with PBS and counted.

### GC mass for evaluation of methylation level of cells

Gas chromatography was done as Rossella describes. In brief, 2.5 micro-gram of DNA was hydrolyzed in 88% of formic acid at 140 °C for 90 min. The samples were evaporated with nitrogen gas. Then 20 macro liters of IS spoliation were added. For Derivatization, we added 50 mL MTBSTFA + 1% TMCS plus 40 mL of acetonitrile and let them react for 30 min at 40 °C. The analysis was performed on Thermo GCMS-QP2000 (Thermo Finnigan). The separation was performed on DB5 (30 m × 0.25 mm × 0.25 μm) with 0.25 μm thickness (Phenomenex). The setups were started at 120 °C for 2 min and then increased by 15 °C per min ratio. The injector temperature was 250 °C in splitless mode. The helium 99.99% was selected as a gas with 1 ml per min velocity. 70 eV was selected for ionization and a 50–400 *m/z* scan was performed. The 250, 280, and 250 C were chosen for the injection duct, ion source, and transfer line. Monitoring the SIM for quantitative analysis of cytosine and 5 mC was used. 254 and 240 *m/z* were mass indices for cytosine. 269, 254, and 240 were used for 5 mC. Xcaliber was used for analysis^41,42^.

### RT-PCR, PCR, and telomere length tests

The extraction of RNA was performed by TRI reagent (Sigma). The RT-PCR was performed with SYBR green by ABI 7500. For validation of RNA quality, agarose gel electrophoresis was performed, and SDS gel electrophoresis was done for PCR and real-time PCR products. Real-time analysis and normalization were performed by QGENE software as mentioned in the MIQE. The Telomere length test was performed as it was reported by RM Cawthon in 2002. In sum, DNA was extracted by TRI reagent according to the manufacturer's protocol. Serial dilution of DNA has been readied and RT-PCR of the samples has been performed by named primer in RM Cawthon^43,44^.

### Immunofluorescence microscopy

The cells were fixed with methanol (Millipore), permeabilized with 0.5% triton × 100 (Sigma), and washed with PBS. The primary antibody was added and then washed with PBS. The secondary antibody was used in 1:200 dilutions. The primary antibodies, including Elf5 (AVIVA System Biology Cat# OAAN02164), Cdx2 (Abcam Cat# ab76541, RRID: AB_1523334), Ssea1 (scbt, Cat# sc-21702) were used. The secondary antibodies were anti-mouse IGg (Sigma-Aldrich, Cat# 62197, RRID: AB_1137649), goat anti-rabbit FITC (Santa Cruz Biotechnology, Cat# sc-2010, RRID: AB_631735), donkey anti-goat Rhodamine (Santa Cruz Biotechnology, Cat# sc-2092, RRID: AB_649000) and for Nanog, the Anti-Nanog-PE, mice (Miltenyi Biotec Cat# 130-104-479, RRID: AB_2652985) were provided. DAPI has been used for staining the nucleus (D9542 SIGMA).

### Embryo transfer

At eight days, cells were trypsinized and relocated to 3.5 cm Petri dishes. After 40 min, the non-attached cells were harvested and relocated to a transfer solution consisting of 10% FBS, 89% DMEM, and 1% antibiotic. Mice were anesthetized with Ketamine. The cells were injected into pseudopregnant NMR mice.

### Ethics and animal rights

All experimental protocols were approved by a Shahid Beheshti University institutional licensing committee. All methods were carried out in accordance with Shahid Beheshti University regulations, and ARRIVE guidelines.

### Statistical analysis

Statistical analysis has been performed with GraphPad Prism 8. T-test and ANOVA tests have been performed as it fitted and P value under 0.05 has been considered as significant.

## Data Availability

All the data generated/analyzed during the study are included in this published article.

## References

[1] Baker CL, Pera MF (2018). Capturing totipotent stem cells. Cell Stem Cell.

[2] Takahashi K, Yamanaka S (2006). Induction of pluripotent stem cells from mouse embryonic and adult fibroblast cultures by defined factors. Cell.

[3] Choi YJ, Lin CP, Risso D, Chen S, Kim TA, Tan MH, Li JB, Wu Y, Chen C, Xuan Z, Macfarlan T (2017). Deficiency of microRNA miR-34a expands cell fate potential in pluripotent stem cells. Science.

[4] Cowan CA, Atienza J, Melton DA, Eggan K (2005). Nuclear reprogramming of somatic cells after fusion with human embryonic stem cells. Science.

[5] Wilmut I, Schnieke A, McWhir J, Kind A, Campbell K (2007). Viable offspring derived from fetal and adult mammalian cells. Cloning Stem Cells.

[6] Makhani K, Ali SM, Yousuf S, Siddiqui S (2015). Therapeutic potential of totipotent, pluripotent and multipotent stem cells. MOJ Cell Sci. Rep..

[7] Harrison S, Sozen B, Christodoulou N, Kyprianou C, Zernicka-Goetz M (2017). Assembly of embryonic and extraembryonic stem cells to mimic embryogenesis in vitro. Science.

[8] Schulz KN, Harrison MM (2018). Mechanisms regulating zygotic genome activation. Nat. Rev. Genet..

[9] Olbrich T, Vega-Sendino M, Tillo D, Wu W, Zolnerowich N, Pavani R, Tran AD, Domingo CN, Franco M, Markiewicz-Potoczny M, Pegoraro G (2021). CTCF is a barrier for 2C-like reprogramming. Nat. Commun..

[10] Xu Y, Zhao J, Ren Y, Wang X, Lyu Y, Xie B, Sun Y, Yuan X, Liu H, Yang W, Fu Y (2022). Derivation of totipotent-like stem cells with blastocyst-like structure forming potential. Cell Res..

[11] Macfarlan TS, Gifford WD, Agarwal S, Driscoll S, Lettieri K, Wang J, Andrews SE, Franco L, Rosenfeld MG, Ren B, Pfaff SL (2011). Endogenous retroviruses and neighboring genes are coordinately repressed by LSD1/KDM1A. Genes Dev..

[12] Ghazimoradi MH, Khalafizadeh A, Babashah S (2022). A critical review on induced totipotent stem cells: Types and methods. Stem Cell Res..

[13] Baker CL, Pera MF (2018). Capturing totipotent stem cells. Cell Stem Cell.

[14] Elhamamsy AR (2016). DNA methylation dynamics in plants and mammals: Overview of regulation and dysregulation. Cell Biochem. Funct..

[15] Jones PA (2012). Functions of DNA methylation: Islands, start sites, gene bodies and beyond. Nat. Rev. Genet..

[16] Uysal F, Akkoyunlu G, Ozturk S (2015). Dynamic expression of DNA methyltransferases (DNMTs) in oocytes and early embryos. Biochimie.

[17] Li Z, Dai H, Martos SN, Xu B, Gao Y, Li T, Zhu G, Schones DE, Wang Z (2015). Distinct roles of DNMT1-dependent and DNMT1-independent methylation patterns in the genome of mouse embryonic stem cells. Genome Biol..

[18] Okano M, Bell D, Haber D, Li E (1999). DNA methyltransferases Dnmt3a and Dnmt3b are essential for de novo methylation and mammalian development. Cell.

[19] Li E, Bestor T, Jaenisch R (1992). Targeted mutation of the DNA methyltransferase gene results in embryonic lethality. Cell.

[20] Wu H, Zhang Y (2014). Reversing DNA methylation: Mechanisms, genomics, and biological functions. Cell.

[21] Farthing CR, Ficz G, Ng RK, Chan CF, Andrews S, Dean W, Hemberger M, Reik W (2008). Global mapping of DNA methylation in mouse promoters reveals epigenetic reprogramming of pluripotency genes. PLoS Genet..

[22] Tsuji-Takayama K, Inoue T, Ijiri Y, Otani T, Motoda R, Nakamura S, Orita K (2004). Demethylating agent, 5-azacytidine, reverses differentiation of embryonic stem cells. Biochem. Biophys. Res. Commun..

[23] Ng RK, Dean W, Dawson C, Lucifero D, Madeja Z, Reik W, Hemberger M (2008). Epigenetic restriction of embryonic cell lineage fate by methylation of Elf5. Nat. Cell Biol..

[24] Boukamp P, Chen J, Gonzales F, Jones PA, Fusenig NE (1992). Progressive stages of" transdifferentiation" from epidermal to mesenchymal phenotype induced by MyoD1 transfection, 5-Aza-2'-deoxycytidine treatment, and selection for reduced cell attachment in the human keratinocyte line HaCaT. J. Cell Biol..

[25] Cho Y, Kim B, Bae H, Kim W, Baek J, Woo K, Lee G, Seol Y, Lee Y, Ku Y, Rhyu I (2017). Direct gingival fibroblast/osteoblast transdifferentiation via epigenetics. J. Dent. Res..

[26] Kharizinejad E, Minaee Zanganeh B, Khanlarkhani N, Mortezaee K, Rastegar T, Baazm M, Abolhassani F, Sajjadi SM, Hajian M, Aliakbari F, Barbarestani M (2016). Role of spermatogonial stem cells extract in transdifferentiation of 5-Aza-2′-deoxycytidine-treated bone marrow mesenchymal stem cells into germ-like cells. Microsc. Res. Tech..

[27] Harris DM, Hazan-Haley I, Coombes K, Bueso-Ramos C, Liu J, Liu Z, Li P, Ravoori M, Abruzzo L, Han L, Singh S (2011). Transformation of human mesenchymal cells and skin fibroblasts into hematopoietic cells. PLoS ONE.

[28] Zaitseva I, Zaitsev S, Alenina N, Bader M, Krivokharchenko A (2007). Dynamics of DNA-demethylation in early mouse and rat embryos developed in vivo and in vitro. Mol. Reprod. Dev..

[29] Wu SC, Zhang Y (2010). Active DNA demethylation: Many roads lead to Rome. Nat. Rev. Mol. Cell Biol..

[30] Huang Z, Yu J, Cui W, Johnson BK, Kim K, Pfeifer GP (2021). The chromosomal protein SMCHD1 regulates DNA methylation and the 2c-like state of embryonic stem cells by antagonizing TET proteins. Sci. Adv..

[31] Jones PA, Taylor SM (1980). Cellular differentiation, cytidine analogs and DNA methylation. Cell.

[32] Taylor SM, Jones PA (1979). Multiple new phenotypes induced in and 3T3 cells treated with 5-azacytidine. Cell.

[33] Tsuji-Takayama K (2004). Demethylating agent, 5-azacytidine, reverses differentiation of embryonic stem cells. Biochem. Biophys. Res. Commun..

[34] Ng RK (2008). Epigenetic restriction of embryonic cell lineage fate by methylation of Elf5. Nat. Cell Biol..

[35] Morgani SM, Brickman JM (2014). The molecular underpinnings of totipotency. Philos. Trans. R. Soc. B Biol. Sci..

[36] Dan J, Rousseau P, Hardikar S, Veland N, Wong J, Autexier C, Chen T (2017). Zscan4 inhibits maintenance DNA methylation to facilitate telomere elongation in mouse embryonic stem cells. Cell Rep..

[37] Ghazimoradi MH, Farivar S (2020). The role of DNA demethylation in induction of stem cells. Prog. Biophys. Mol. Biol..

[38] Yang F, Huang X, Zang R, Chen J, Fidalgo M, Sanchez-Priego C, Yang J, Caichen A, Ma F, Macfarlan T, Wang H (2020). DUX-miR-344-ZMYM2-mediated activation of MERVL LTRs induces a totipotent 2C-like state. Cell Stem Cell.

[39] Zuo F, Jiang J, Fu H, Yan K, Liefke R, Zhang J, Hong Y, Chang Z, Liu N, Wang Z, Xi Q (2022). A TRIM66/DAX1/Dux axis suppresses the totipotent 2-cell-like state in murine embryonic stem cells. Cell Stem Cell.

[40] Gonzalo S, Jaco I, Fraga MF, Chen T, Li E, Esteller M, Blasco MA (2006). DNA methyltransferases control telomere length and telomere recombination in mammalian cells. Nat. Cell Biol..

[41] Okamoto Y, Yoshida N, Suzuki T, Shimozawa N, Asami M, Matsuda T, Kojima N, Perry AC, Takada T (2016). DNA methylation dynamics in mouse preimplantation embryos revealed by mass spectrometry. Sci. Rep..

[42] Rossella F, Polledri E, Bollati V, Baccarelli A, Fustinoni S (2009). Development and validation of a gas chromatography/mass spectrometry method for the assessment of genomic DNA methylation. Rapid Commun. Mass Spectrom..

[43] Cawthon RM (2002). Telomere measurement by quantitative PCR. Nucleic Acids Res..

[44] Farivar S, Ghazimoradi MH (2019). DNA and RNA extraction from low amount of blood volume. Forensic Sci. Int..

